# Annotations

**Published:** 1910-07-09

**Authors:** 


### Annotations.

### European Diminution in Tuberculosis.

In a demographical survey published at Berlin and dedicated to the International Exhibition of Hygiene to be held next year at Dresden, K. E. Lingner shows statistical curves which yield the following interpretations. Most European countries, especially England and Wales, and also Germany, show a lessening in the mortality from consumption. This lessening was, however, in progress just as rapidly before the discovery of the tubercle bacillus as after it, at all events in the case of England, which has by far the oldest (since 1845) statistics. Switzerland, Austria, and Italy have made very little advance, France is stationary, while Iceland, Norway, and Finland actually show an increase in deaths from pulmonary tuberculosis. The curve of all cases of tuberculosis generally follows that of consumption, owing to the preponderance of that form of the disease. In Germany it is lowest in Prussia and Saxony, highest in Bayern, Baden, Hesse, and Alsace-Lorraine. The industrial cities, curiously enough, show the greatest diminution in tuberculosis mortality. As regards non-pulmonary forms, it is a striking fact that no improvement is to be noted in any country, except perhaps a very gradual reduction in England and Wales. Improvement in exactitude of diagnosis surely cannot account for this result.

### The Physical Menace of the Negro.

We, who rarely if ever come across a black man, have little idea of the negro problem which has troubled the minds of all thinking Americans for the past few years, a problem which, if we are to believe the statements of Dr. Murrell, who writes in the *Journal of the American Medical Association*, is yearly becoming more acute. The author, who is connected with the University College of Medicine at Richmond, has had exceptional opportunities of studying American negroes from the medical standpoint, and finds that their bodies have deteriorated in proportion as their minds have developed. What strikes him most, as the result of his researches, is the truly awful prevalence of syphilis among the members of the black community. The well-known susceptibility of negroes to all forms of tuberculosis, which results in large numbers of them falling victims to phthisis, is quite eclipsed by their susceptibility to syphilis, and he estimates that for every tuberculous there are twenty syphilitic negroes. He predicts that in fifty years' time an untainted negro will be something of a medical freak. Again, in the registration area the black death rate stands at 30.2 per thousand, as compared with 17.3 among the whites. In the last fifty years negro insanity has increased by a thousand per cent.! Add to these facts the ravages of the cocaine habit, to which the black man is notoriously addicted, and to the indulgence in which habit are due many of the cases of rape of white women by negroes; at the same time consider also the obvious drawbacks of miscegenation, and the problem ceases to be a local and becomes a national one. The cause of this state of things is not far to seek, and is best described in the author's own words: "When the curse of slavery, for it was a curse, was removed from the white man's shoulders, misguided theorists transferred it under the name of freedom to the unready negro, and it crushed him. Whatever the motive that guided the pen which decreed absolute suffrage, it stands as one of the world's great tragedies, for now the negro was free, not to live, but to die, and he took advantage of his freedom. He was free, indeed—free as the birds of the air, free to get drunk with cheap political whiskey and to shiver in the cold because his scanty savings went to purchase flashy and flimsy garments—free never to bathe, and to sleep in hovels where God's sunlight and air could not penetrate—absolutely free to gratify his every sexual impulse; to be infected with every loathsome disease and to infect his ready and willing companions—and he did it—he did it all. The result is the negro of 1910, the negro of to-day."

### The Wisdom of the Ancients.

The sacred books of most religions contain many excellent sanitary precepts which, while based merely on an intelligent observation of Nature, are often startlingly accurate in their effectiveness, as the following article will show. The *Journal of Tropical Medicine and Hygiene* points out that, so far from being contrary to the religion of any considerable section of the population of India, rat-killing is actually enjoined in their sacred books. According to Hindu ideas, the Vedas stands on even higher authority than is claimed by Christians for their Bible, as they are said not to be merely inspired but to have existed prior to the beginning of time. The following is a translation from Book VI, verse 50, of the Atharva-Veda:—

> "Destroy the rat, the mole, the boring beetle; cut off their heads, O asvins.
> Bind fast their mouths; let them not eat our barley; so guard ye twain our growing corn from danger.
> Hearken to me, lord of the female borer, lord of the female grub! Ye rough-toothed vermin.
> Whate'er ye be, dwelling in woods, and piercing, we crush and mangle all those piercing insects."

The journal goes on to remark that the destruction of the flea and mosquito, both piercing insects, is clearly enjoined as well as that of the rat, and it is certain that if the Veda could be literally obeyed by its followers, plague, malaria, and filariasis would be promptly abolished from India, and trypanosomes cease to destroy its cattle. This passage in the original Sanscrit with a translation into the modern vernacular should be incorporated into a leaflet for general distribution, and introduced into any book prepared for instruction in elementary hygiene for the use of vernacular schools.

